# Tracing mother-infant transmission of bacteriophages by means of a novel analytical tool for shotgun metagenomic datasets: METAnnotatorX

**DOI:** 10.1186/s40168-018-0527-z

**Published:** 2018-08-20

**Authors:** Christian Milani, Eoghan Casey, Gabriele Andrea Lugli, Rebecca Moore, Joanna Kaczorowska, Conor Feehily, Marta Mangifesta, Leonardo Mancabelli, Sabrina Duranti, Francesca Turroni, Francesca Bottacini, Jennifer Mahony, Paul D. Cotter, Fionnuala M. McAuliffe, Douwe van Sinderen, Marco Ventura

**Affiliations:** 1 Laboratory of Probiogenomics, Department of Chemistry, Life Sciences and Environmental Sustainability, University of Parma, Parco Area delle Scienze 11a, 43124 Parma, Italy; 2 APC Microbiome Ireland, University College Cork, Cork, Ireland; 3 School of Microbiology, University College Cork, Cork, Ireland; 4 UCD Perinatal Research Centre, School of Medicine, University College Dublin, National Maternity Hospital, Dublin, Ireland; 5 Teagasc, Moorepark Food Research Centre, Fermoy, Co., Cork, Cork, Ireland; 6 GenProbio srl, Parma, Italy; 7 Microbiome Research Hub, University of Parma, Parma, Italy

**Keywords:** Gut microbiota, Metagenomics, Metagenome, Virome, Gastro intestinal tract, Vertical transmission

## Abstract

**Background:**

Despite the relevance of viral populations, our knowledge of (bacterio) phage populations, i.e., the phageome, suffers from the absence of a “gold standard” protocol for viral DNA extraction with associated in silico sequence processing analyses. To overcome this apparent hiatus, we present here a comprehensive performance evaluation of various protocols and propose an optimized pipeline that covers DNA extraction, sequencing, and bioinformatic analysis of phageome data.

**Results:**

Five widely used protocols for viral DNA extraction from fecal samples were tested for their performance in removal of non-viral DNA. Moreover, we developed a novel bioinformatic platform, METAnnotatorX, for metagenomic dataset analysis. This in silico tool facilitates a range of read- and assembly-based analyses, including taxonomic profiling using an iterative multi-database pipeline, classification of contigs at genus and species level, as well as functional characterizations of reads and assembled data. Performances of METAnnotatorX were assessed through investigation of seven mother-newborn pairs, leading to the identification of shared phage genotypes, of which two were genomically decoded and characterized.

METAnnotatorX was furthermore employed to evaluate a protocol for the identification of contaminant non-viral DNA in sequenced datasets and was exploited to determine the amount of metagenomic data needed for robust evaluation of human adult-derived (fecal) phageomes.

**Conclusions:**

Results obtained in this study demonstrate that a comprehensive pipeline for analysis of phageomes will be pivotal for future explorations of the ecology of phages in the gut environment as well as for understanding their impact on the physiology and bacterial community kinetics as players of dysbiosis and homeostasis in the gut microbiota.

**Electronic supplementary material:**

The online version of this article (10.1186/s40168-018-0527-z) contains supplementary material, which is available to authorized users.

## Background

The establishment of Next Generation Sequencing (NGS) technologies has facilitated explorations into the ecology and functionality of microorganisms living in complex communities [1]. Notably, a substantial portion of these research efforts has focused on the characterization of prokaryotes colonizing humans, being microbiota members that reside in various body sites, investigations that have clearly revealed the existence of an intimate relationship between these microbial populations and their host [2]. In this context, bacteria colonizing the gastro-intestinal tract have been described as a “forgotten organ” based on their understudied, yet key roles in a wide range of aspects of animal physiology, including the development, metabolism, and functionality of the immune system [3, 4].

Despite the scientific interest in the bacterial component of the gut microbiota, current knowledge on the associated (bacterio) phage populations, i.e., the phageome, is very limited. These bacterial viruses are believed to play an important role in influencing the ecology of prokaryotes, e.g., by modulating population dynamics and catalyzing horizontal gene transfer events [5], although knowledge on their prevalence, diversity, and specific functionalities is still in its infancy. In this context, only a limited number of studies have evaluated the functional role of phages in the gastrointestinal tract (GIT), the majority of which provide a descriptive profiling of the viral population in saliva or fecal samples [6–12]. This rather naïve view of phage ecology in the GIT reflects the very limited exploration of the role, if any, of phages in the development and evolution of common gut diseases, with studies focusing mainly on inflammatory bowel diseases (IBD), such as Crohn’s disease (CD) and ulcerative colitis (UC) [13, 14]. This knowledge gap can primarily be attributed to the lack of a comprehensive experimental pipeline for metagenomic analyses of viral populations that ideally should include an efficient and reliable protocol for viral DNA extraction and purification, as well as bioinformatic tools for phageome data management, processing and associated analysis. In fact, while a range of optimized protocols for extraction of phage DNA have been published [15, 16], their efficiency has not yet been comparatively assessed, primarily because the tools that are currently available for the analysis of phage metagenomic datasets rely on simple homology searches against a single viral database [17, 18]. Thus, the absence of data regarding other components of the metagenomic dataset, i.e., archaea, bacteria, and eukaryotes, does not permit an accurate evaluation of the viral DNA retrieved from an environmental sample. Moreover, the lack of available tools for efficient phageome assembly and subsequent functional interrogation and taxonomic classification of generated contigs prevents identification and reconstruction of the complete genome of free phage particles. Altogether, these limitations underline the need for a thorough assessment of available methodologies for phageome analysis, with particular focus on the identification of the viral DNA extraction protocol providing the lowest relative abundance of exogenous DNA, as well as definition of a comprehensive bioinformatic pipeline for phylogenetic and genomic characterization of the viral population.

For these reasons, the objective of the current report was to develop a start-to-finish protocol to cover phageome analysis from DNA extraction of fecal samples all the way to sequence data processing and database interrogations. We therefore performed a comparative analysis of the five most widely employed protocols for viral DNA extraction and purification from fecal samples, coupled with an in-depth evaluation of the generated sequences by means of a novel viral metagenomics analysis platform, which we called METAnnotatorX. This bioinformatics analysis platform supports a wide range of read- and assembly-based analyses using a multi-database, homology-based search approach to explore the viral, archaeal, bacterial, and eukaryotic biodiversity within a generated sequence dataset from a given sample.

In order to provide an example of the functionality offered by analysis of phageomes, the optimal identified protocol for viral DNA extraction and METAnnotatorX was employed so as to profile phageomes of fecal samples collected from seven mothers and their corresponding infants. Results allowed the detection of mother-to-infant vertical transmission of phages, two of which were also genomically decoded and annotated.

## Methods

### Ethical statement and sample collection

The study protocol was approved by the National Maternity Hospital Dublin ethics committee, and informed written consent for fecal sample collection and associated microbiological analyses was obtained from all participants or their legal guardians.

### Virus-like particle (VLP) isolation and DNA extraction

#### Extraction protocols 1A, 1B, and 1C

0.5 g of fecal material was suspended in 45 ml of sterile SMG (sodium chloride magnesium sulphate) buffer (200 mM NaCl, 10 mM MgSO_4_, 50 mM Tris-HCl (pH 7.5), 0.01% gelatin) and homogenized in filter bags for 2 min at medium speed. The resultant solution was then incubated on ice for 1 h for virus-like particle (VLP) desorption. Samples were then centrifuged at 5000×*g* for 45 min at 4 °C. Supernatants were recovered and large particulates were removed using Whatman glass microfibre filters (Sigma-Aldrich, St. Louis, MO, USA). A second centrifugation step of 5000×*g* for 45 min at 4 °C was performed; the supernatant was then collected and, in the case of protocol 1A, used for VLP precipitation through supplementation with 10% PEG 6000 (Sigma-Aldrich, St. Louis, MO, U.S.A.) at 4 °C overnight. In contrast, in the case of protocol 1B, the supernatant was first subjected to 0.45-μm filtration (all filters obtained from Sarstedt, Numbrecht, Germany), while for protocol 1C, the supernatant was subjected to 0.45-μm filtration, followed by a 0.2-μm filtration, before precipitation of VLPs. PEG-precipitated VLPs were collected by centrifugation at 25000×*g* for 45 min at 4 °C. The resulting VLP-containing pellets where then re-suspended in 400 μl SMG buffer at 4 °C. The sample was DNase treated with 10 U ml^−1^ DNase I (Roche, Basel, Switzerland) for 1 h at room temperature with subsequent inactivation performed by heat treatment at 75 °C for 10 min. Viral DNA was then extracted using the Norgen Phage DNA isolation kit (Norgen Biotek Corp., Ontario, CA) according to the manufacturer’s instructions.

#### Extraction protocols FD and DTT

0.5 g of fecal material was suspended in 1.2 mL of SMG buffer by vortexing for 2 min. The resultant solution was then incubated on ice for 1 h. Following incubation, a centrifugation step of 2500×*g* for 5 min at 4 °C was performed. The supernatant was then centrifuged again at 5000×*g* for 15 min at 4 °C. The supernatant was retained, and dithiothreitol (DTT) (Promega, Madison, WI, USA) was added to a final concentration of 6.5 mM and incubated for 1 h at 37 °C. In the FD protocol, this DTT treatment was absent. The resultant solution was then filtered employing a 0.45-μm filter. The sample was DNase treated with 10 U ml^−1^ DNase I (Roche) for 1 h at room temperature with subsequent inactivation performed by heat treatment at 75 °C for 10 min. Viral DNA was then extracted using the Norgen Phage DNA isolation kit according to the manufacturer’s instructions. DNA concentrations were quantified using the Qubit Fluorometer and Qubit dsDNA HS Assay Kit (Life Technologies, Bleiswijk, Netherlands).

### Shotgun metagenomics sequencing and analysis

DNA was fragmented to 550–650 bp using a BioRuptor machine (Diagenode, Belgium). Samples were prepared following the TruSeq Nano DNA Sample Preparation Guide (Part#15041110Rev.D). Sequencing was performed using an Illumina NextSeq 500 sequencer with NextSeq Mid Output v2 Kit chemicals (Illumina Inc., San Diego, CA 92122, USA). Read- and assembly-based analyses were performed using the METAnnotatorX bioinformatic platform described below in this manuscript. Mapping of reads on nucleotide sequences was performed using the software BowTie2 [19] and retrieval of mapping or non-mapping reads was performed using the Sequence Alignment/Map tools (SAMtools) 43 [20].

### METAnnotatorX

The METAnnotatorX bioinformatics platform described in this manuscript performs a range of in silico taxonomic and functional analyses of both reads and contigs assembled from shotgun metagenomics datasets. Details are reported in the “Results and Discussion” section while the default METAnnotatorX settings, used for all analyses reported in this manuscript, are listed in Additional file 1: Table S1.

## Results and discussion

### Comparative evaluation of various protocols for viral DNA extraction and purification

Virome protocol analyses commonly consist of the isolation of virus-like particles (VLPs) from a fecal sample followed by extraction of the genetic material from these VLPs, prior to further analysis of the obtained genetic material by means of shotgun sequencing approaches [21–23]. Published protocols for VLP isolation from fecal samples all involve homogenization of fecal samples in a buffer, followed by centrifugation steps to remove bacteria and large particles, with a subsequent filtration step. Total nucleic acid can then be isolated from the resulting filtrate following a DNase treatment to remove bacterial DNA contamination [21–24].

Despite several attempts to optimize protocols for fecal VLP extraction (5, 6), a “gold standard” protocol has yet to be developed and to be accepted by the scientific community. A trial of an optimized PEG-precipitation method (Route 5 from [15] termed protocol 1A here) was undertaken with some modifications. Following sample homogenization, an incubation step on ice was included to encourage VLP desorption [25]. The other key modification of the protocol represents the omission of a CsCl density gradient centrifugation step as this has been shown to have a detrimental effect on phage infectivity [15] and can influence retrieved information on community composition by introducing a bias against certain phages [16]. Omission of the CsCl step is believed to lead to a more faithful representation of community composition, yet at the expense of a reduced efficiency of bacterial DNA removal [16]. To counteract this, we tested dead-end filtration steps, where protocol 1A lacked such a filtration step, protocol 1B included a 0.45-μm filtration step, whereas samples processed using protocol 1C were subjected to 0.45 μm followed by a 0.2-μm filtration, (in all protocols) prior to PEG precipitation. Furthermore, it was determined through a phage spiking experiment that PEG removal by buffer exchange was inefficient and in fact caused loss of phages during subsequent centrifugation (data not shown); therefore, DNA extraction was directly performed on the resuspended PEG-precipitated VLPs.

In addition to these protocols, two further methods from literature, namely the FD (termed here as 1D) and DTT (termed here as 1E) methods described by Kleiner et al. [16], were assessed. These PEG precipitation-based protocols require simple homogenization of the sample followed by filtration, DNase treatment, and DNA extraction, with the only difference between the two being a dithiotreitol treatment to degrade fecal mucus in the 1E protocol. In the current study, we modified these two protocols by the inclusion of a VLP desorption step and adjustment of the initial sample size. DNA yields were comparable when applying these five protocols on the same fecal sample, except in the case of protocol 1A, which yielded approximately four times more DNA compared to the other assessed protocols (Table 1). This was presumably due to the presence of host-derived DNA contaminating the viral DNA due to the lack of filtration and/or a density gradient centrifugation step. In terms of practical and experimental advantages, the 1D and 1E methods are vastly preferable to the 1A, 1B, and 1C methods in terms of execution time, with protocol completion achievable within 1 day as compared to 2 days, while also offering the advantage of a considerably shorter “hands-on” procedure (Table 1).

**Table 1:** Overview of viral DNA extraction protocols

Protocol	1A	1B	1C	1D	1E
Total DNA yield (ng)	58.3	8.2	10.2	15	10.2
Sample throughput (no. of samples processed simultaneously)	15–20	15–20	15–20	15–20	15–20
Protocol duration (days)	2	2	2	1	1
Hands-on time (hours)	10	10	10	6	7

### Development of a comprehensive bioinformatic pipeline for analysis of shotgun metagenomic datasets

**Fig. 1:**
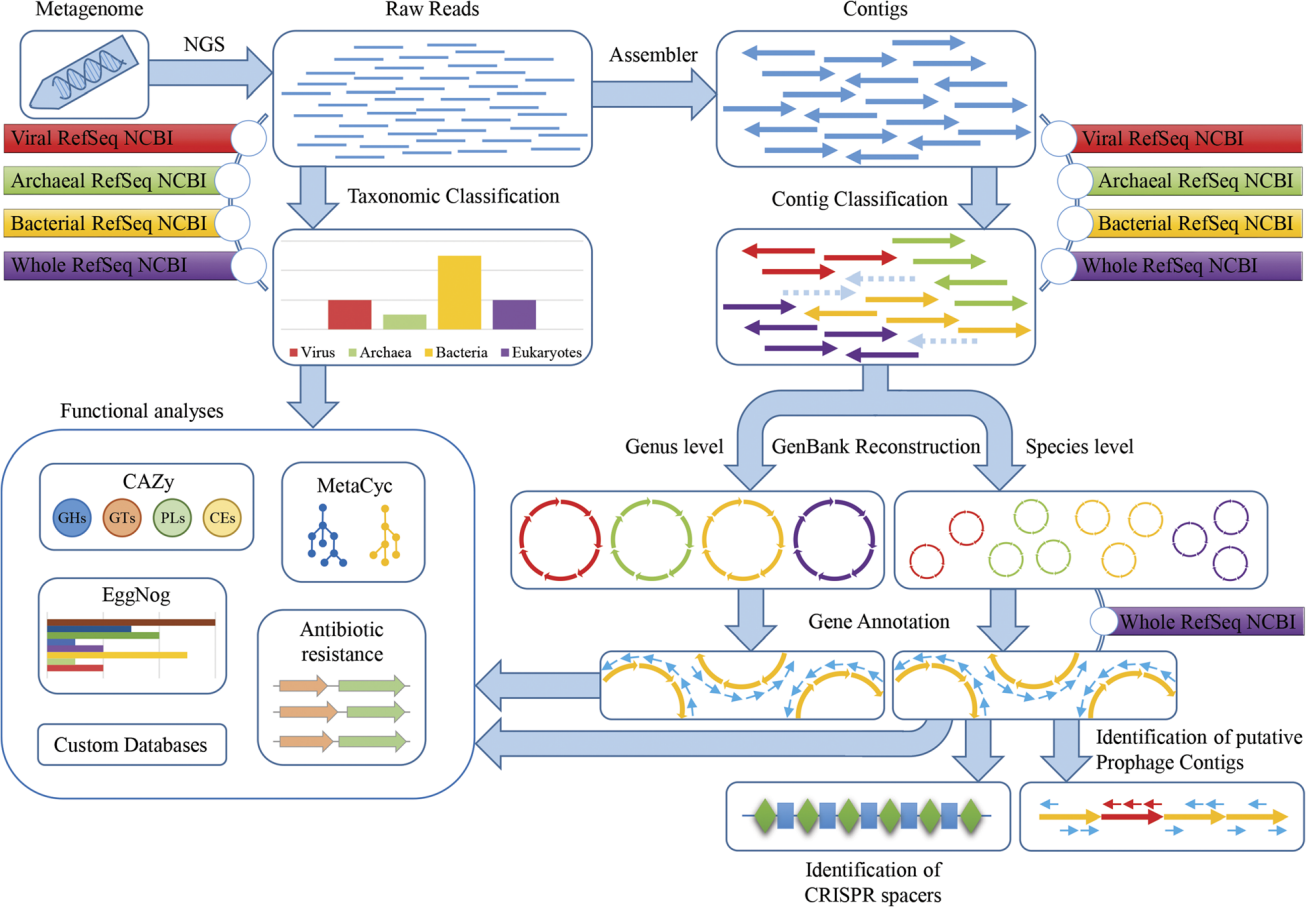
Schematic representation of the automated analyses performed by METAnnotatorX. Raw reads obtained from NGS sequencing can be directly used as input data for a range of read- and assembly-based analyses

A large proportion of the current, publicly available tools for analysis of (bacterio) phage populations, i.e., the phageome, relies on alignment against a single viral database to obtain taxonomic assignment of reads or pre-assembled contigs [17, 18]. This approach is very limiting since shotgun metagenomics datasets are mainly employed for taxonomic surveys, though such datasets may be able to generate novel information regarding genomic structure, functionality and host-specificity of identified phages. To fill these gaps, we developed a comprehensive bioinformatic platform, referred to here as METAnnotatorX, which performs a variety of analytical steps applied to a given shotgun metagenomic dataset. METAnnotatorX not only performs taxonomic and functional profiling of the reads, but also allows assembly and phage genome reconstruction, open reading frame identification, and annotation (Fig. 1). Moreover, the developed pipeline is able to analyze the read pools corresponding to archaea, bacteria, and eukaryotes through iterative classification steps that exploit specific databases for viruses, bacteria, archaea, and eukaryotes. Notably, viruses are classified at the family and species level, while bacteria, archaea, and eukaryotes are classified at the genus and species level. Thus, the pipeline can be exploited not only to perform a comprehensive analysis of viromes, but also of shotgun metagenomic datasets that include bacterial, archaeal, and eukaryotic data (Fig. 1). METAnnotatorX is provided pre-installed in a virtual machine running Ubuntu 16.04.3 (http://probiogenomics.unipr.it/pbi/index.html). A graphic installation interface guides the user through a small number of steps for third party software installation and database downloading, which are necessary to install METAnnotatorX.

The main graphic interface of the METAnnotatorX software allows selection of input dataset (s), output folder, and analysis steps (Fig. 1 and Additional file 1: Figure S1). Moreover, a configuration file provides the user with an option to modify a range of associated analysis parameters, such as the number of computing cores or databases to be used and specific cut-offs (Additional file 1: Figure S1). Outputs are provided as tabular files ready to be imported in spreadsheet software or as GenBank files in the case of assembled and annotated sequences (Additional file 1: Figure. S1).

METAnnotatorX provides an innovative approach for taxonomic profiling of reads that relies on four consecutive read annotation steps querying four NCBI databases, including the Viral RefSeq, Archaeal RefSeq, bacterial RefSeq, and the whole RefSeq for eukaryote classification (Fig. 1). Notably, hits against the viral RefSeq are in default mode given the maximum priority, followed by archaeal RefSeq, bacterial RefSeq, and the whole RefSeq, so as to guarantee high sensitivity towards viral and archaeal profiling and to avoid annotation of viral reads as archaeal or bacterial in case of prophages that constitute part of prokaryotic genomes. In this regard, it is noteworthy that read-based metagenomic approaches cannot distinguish between reads corresponding to free viral particles and reads belonging to prophage genomes. Thus, efficient removal of non-viral DNA during DNA extraction is fundamental to minimize misclassification of prophages as free viral particles when analyzing phageomes. RefSeq databases are non-redundant datasets built from the sequence data available in the archival database GenBank, and each RefSeq record represents a synthesis of information obtained from GenBank records with identical sequences [26, 27].

It is also worth mentioning that the viral RefSeq database was selected as the default database for viral taxonomic classification since all its entries are genes predicted from manually revised and validated viral genomes. Although the viral RefSeq database is continually expanded and updated (at the time of writing of this manuscript), it encompasses 7485 genomes, whereas the GenBank viral database includes 5530 additional non-revised genomic sequences, thus totaling 13,015 genomes. METAnnotatorX was therefore developed to offer the possibility to interrogate the GenBank viral database as an alternative to the Viral RefSeq database if the user wants to maximize the sensitivity of the analysis while reducing specificity. Moreover, the user can request interrogation of alternative databases in the setting file. Notably, the header of fasta entries must be formatted as those included in the NCBI RefSeq database. In this context, external databases such as the recently published VirSorter [28] and IMG/VR databases [29] may represent useful alternatives. Nevertheless, due to the exponential increase of metagenomic data, such databases require constant updating as performed by NCBI for RefSeq databases.

The user can also choose to perform functional classification analyses of the reads using custom databases for METAnnotatorX that can be downloaded and updated using a script available in the virtual machine. These analyses permit retrieval of (i) COG functional category profiles as based on the EggNog nomenclature [30]; (ii) carbohydrate-active enzymes, i.e., the glycobiome, based on CAZy database nomenclature [31]; and (iii) metabolic pathways based on the MetaCyc classification [32] (Fig. 1).

Furthermore, shotgun metagenomic datasets can also be employed for metagenomic assembly using SPADES software [33] (Fig. 1). Notably, contigs > 5000 nucleotides are taxonomically classified by means of a novel in silico protocol, which taxonomically categorizes encoded ORFs following a multi-step approach, as described above for reads. The contigs are then classified with the most frequent taxonomy observed among genes encoded by each contig. Subsequently, the user can request the generation of GenBank files with annotated ORFs comprised of all contigs that share the same taxonomy at bacterial genus/viral family or species level (Fig. 1). ORFs are annotated based on the MEGAnnotator pipeline for accurate functional assignment [34]. Furthermore, each contig pool which corresponds to a taxonomic rank can be functionally profiled as indicated above.

Additional analyses offered by METAnnotatorX encompass host prediction based on the CRISPRdb [35], as well as evaluation of the relative abundance and taxonomic profile of genes collected in user-provided databases, and identification of putative (pro) phage genomes without homologs in the NCBI Viral RefSeq database through screening of bacterial contigs for those encoding ORFs typically found in genomic modules of phages (Fig. 1).

A comprehensive manual details the pipeline followed by each analysis offered by METAnnotatorX, including software and default cut-off values used (http://probiogenomics.unipr.it/pbi/index.html).

At the time of writing, we could not compare METAnnotatorX with the two available online tools for phageome analysis, i.e., VIROME [17] and MetaVir 2 [18] using a test dataset of known viral composition, due to limitations regarding input data or saturation of storage and computing capacities (details can be found in the Additional file 1). Nevertheless, we performed re-analysis of a dataset already processed with MetaVir2 that can be downloaded from the MetaVir2 website (Additional file 1: Table S2). In this context, comparison of the results retrieved through analysis of these datasets using MetaVir2 and METAnnotatorX revealed that METAnnotatorX is able to detect and classify a higher number of viral taxa (Additional file 1: Table S2). Notably, differences may be attributable to the more updated database and improved pipeline exploited by METAnnotatorX.

### In silico comparative analysis of shotgun metagenomics data obtained from the five tested protocols for viral DNA extraction and purification

In order to reconstruct a detailed overview of the performance of the five tested protocols for double-stranded viral DNA purification, i.e. 1A, 1B, 1C, 1D and 1E, the same infant fecal sample was processed using these five distinct DNA isolation procedures. The obtained DNA was then subjected to Illumina paired-end sequencing. Subsequently, METAnnotatorX was employed for analysis of a sub-sample that consisted of 500,000 randomly selected reads of the total read pool obtained for each viral DNA purification protocol.

**Fig. 2:**
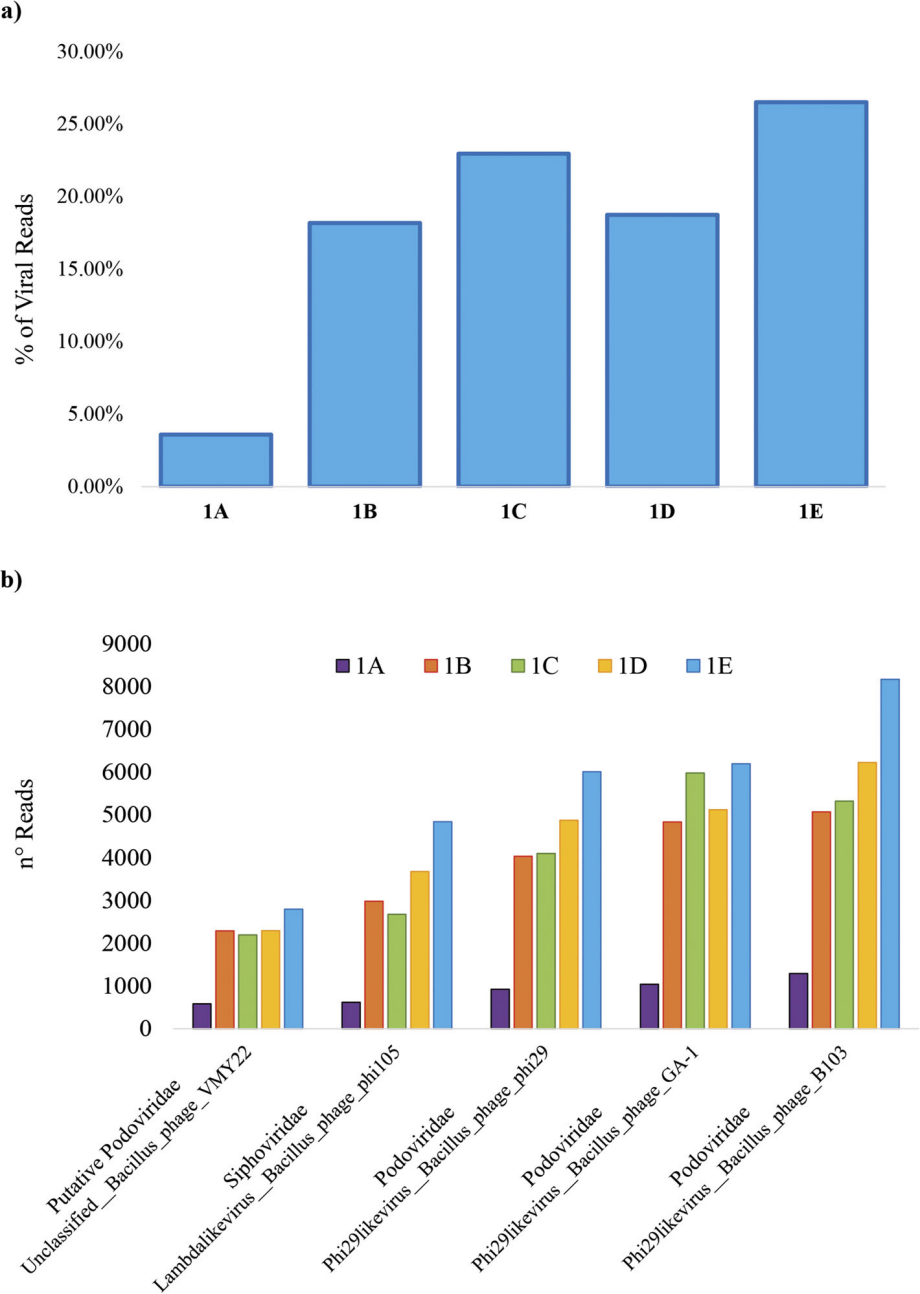
Evaluation of non-viral DNA removal performances through analysis of viral DNA extracted from the same fecal sample using five different protocols. **a** The percentage of viral DNA detected through taxonomic classification of reads corresponding to coding regions. **b** The number of reads retrieved for the five most abundant viral taxa using the five different protocols

Remarkably, read-based taxonomic profiling of the normalized datasets revealed that protocol 1E provides the best performance in terms of removal of non-viral DNA, i.e. the total relative abundance of reads not profiled as viral, in comparison to the other tested protocols (Fig. 2). Moreover, we evaluated the efficiency of recovered viral DNA obtained from the five most abundant viral taxa profiled across all the five datasets (Fig. 2), encompassing both *Siphoviridae* and *Podoviridae* viral families. This was performed through mapping of reads obtained for each sample on the assembled contigs classified as the viral taxa listed in Fig. 2. Notably, evaluation of the number of mapped reads confirmed the superior performance of protocol 1E for all five viral taxa analyzed and demonstrated the absence of a species-specific bias in phage DNA enrichment.

To confirm the observed performances in non-viral DNA removal, the five protocols were used to perform duplicate extractions from an additional human fecal sample. Notably, the obtained results confirmed the superior performance of protocol 1E and did not reveal any biases in the duplicates (Additional file 1: Figure S2).

Overall, the 1E protocol yielded the best results both in terms of execution time (Table 1) and removal of non-viral DNA. Thus, this protocol to isolate and analyze double-stranded viral DNA was employed for processing of subsequent phageomes sequenced in this study. It should be noted that, since we did not include a multiple-displacement amplification (MDA) step in our pipeline, ssDNA viruses were not assessed (yet this can easily be remedied by the inclusion of such an MDA step).

### Evaluation of the sensitivity and specificity of phage classification as performed by METAnnotatorX

An artificial sample of 500,000 reads was constructed using random reads corresponding to the virome of a human adult fecal sample with the addition of decreasing percentages of reads obtained from shotgun sequencing of C2 and 936 *Lactococcus* phages, as outlined in Additional file 1: Table S3. Notably, our findings showed that METAnnotatorX is able to accurately reconstruct the composition of the artificially composed sample, with limited discrepancies (Additional file 1: Table S3).

### Identification of contaminants

The amount of viral DNA extracted from environmental samples may be of very low abundance, in particular when performing viral DNA extraction from samples with very limited bacterial colonization, e.g., meconium samples from newborns. This not only represents an issue for library preparation and sequencing yields but may also cause biases induced by environmental contamination. In fact, if the amount of viral DNA retrieved from a sample is limited, even the presence of a very low quantity of contaminating DNA is expected to result in the presence of a high relative contaminant level of sequencing reads in a given dataset.

In order to identify and remove contaminant DNA in the phageome datasets used in this study, the genome align tool MAUVE [36] was exploited to perform cross-alignment of contigs obtained from the metagenomic datasets using METAnnotatorX. Interestingly, we observed that the five infant samples, which represent the first stool samples of these neonates following birth (i.e. the meconium), used for evaluation of mother-infant vertical transmission of phages (discussed below) share identical contigs (Additional file 1: Figure S3). ORF prediction and functional annotation of these contigs led to the reconstruction of the complete genome of phages extensively studied in our laboratory [37, 38]. Thus, we proceeded to map all datasets included in this study (see above and below for details) to these apparently contaminating contigs using a 99% identity cut-off in order to remove the reads corresponding to these putative contaminants. This cut-off was chosen to allow mapping of reads identical to the backbone, while permitting the 1% error rate that affects Illumina sequencing [39]. Moreover, the DNA extraction kit was identified as the primary source of contaminants and measures were taken to minimize such contamination, including the use of dedicated kits for fecal virome studies [40] and performing DNA isolation in laminar flow hoods [41]. However, in samples with low DNA abundance, the potential for DNA contamination remains significant, and it is therefore strongly recommended to perform routine sequencing of sham controls so as to monitor and identify DNA contaminants originating from the lab environment [42]. In this context, a newly acquired DNA extraction kit was used to process a sham sample, resulting in 46,269 quality-filtered reads, representing 4.6% of the target sequencing depth of 1,000,000 reads. Moreover, assembly of these data did not produce any contigs, thus indicating that the retrieved reads represent the sequencing background, i.e., sequencing and demultiplexing errors performed by the Illumina sequencer [39]. It is worth mentioning that while the latter approach is effective in the removal of contaminants that can be assembled into contigs, it is not possible to efficiently detect non-viral DNA present at low abundance using a read-based approach. Thus, prevention of DNA contamination should be considered critical in virome studies, particularly when analyzing samples with a low viral load.

Notably, the presence of contaminant DNA from the lab environment seems to be a common issue in published phageome studies, as evidenced by MAUVE genomic alignment of contigs assembled from datasets sequenced in one of the largest infant phageome studies [22] and available in the NCBI SRA database (https://www.ncbi.nlm.nih.gov/sra). Interestingly, genome alignment of contigs assembled using METAnnotatorX from 12 random datasets revealed the presence of sequences taxonomically related to the *Pseudomonas* genus that are shared and show identity > 99% by most of the phageomes (Additional file 1: Figure S4). Notably, if a cross check of sequences assembled from unrelated samples processed in the same lab reveals contigs with high identity, they may represent contaminants from the environment. Thus, these contigs should be carefully evaluated and, if they are shown to represent contaminating sequences, be removed from such datasets.

### Evaluation of mother-infant transmission of phages

**Fig. 3:**
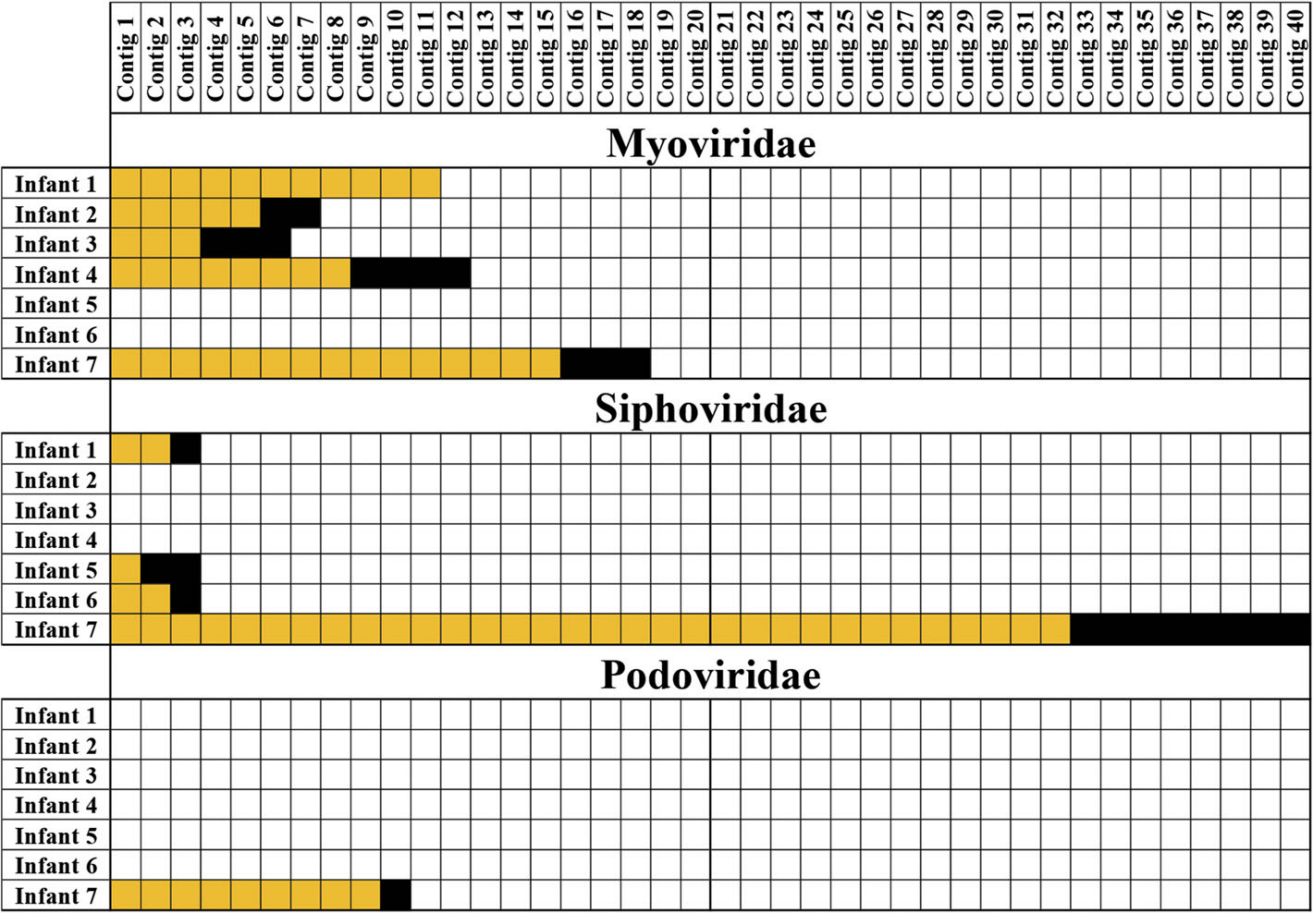
Identification of vertical transmission events of phages. For each of the seven enrolled infants, the assembled viral contigs > 5000 bp were used as backbone for stringent mapping of sequencing reads obtained from their mothers. In case mapping reads were observed, the contig was either colored in yellow or in black

To demonstrate the potential for a comprehensive pipeline for in depth analysis of phageomes, the 1E extraction protocol and METAnnotatorX platform were employed in combination for the analysis of fecal samples collected from seven mothers and their corresponding newborn infants. In total, 14 fecal samples were collected, corresponding to seven mothers sampled at 34 weeks of gestation and meconium samples of their corresponding offspring. Viral DNA was extracted by means of the 1E protocol and sequenced with Illumina technology, aimed at achieving an output of 10 million reads for the meconium samples and 25 million reads for fecal samples of mothers. Shotgun sequencing produced a total of 148,797,588 reads, ranging from 238,288 to 34,105,775 reads (Additional file 1: Table S4). Notably, a high variability of sequencing yield was expected despite normalization of DNA used for library preparation, with those samples that encompass a very low virus load (i.e. meconium). The obtained datasets were processed with METAnnotatorX in order to classify the viral, archaeal, bacterial, and eukaryotic reads (Additional file 1: Figure S5). A complete profile of the archaeal and bacterial viral population is reported in Additional file 2. The obtained read-based taxonomic profiles revealed the presence of common viral taxa in each mother-infant pair (Table 2). To evaluate if the latter observation is due to sharing of the same phage genotypes, METAnnotatorX was employed for taxonomic assignment of contigs reconstructed from the infant datasets. Subsequently, the retrieved phage contigs were used as backbones for mapping of the reads constituting the dataset of the corresponding mother (Fig. 3). To avoid false positives, mappings were performed using a stringent identity cut-off of 99%. As reported above, a 99% cut-off was chosen to allow mapping of reads that are identical to the backbone while permitting the 1% error rate, which is imputable to Illumina sequencing [39]. Notably, for each mother-infant pair, reads of the mother’s phageome were mapped on multiple phage contigs reconstructed from the corresponding infant, thus suggesting a vertical route for phageome transmission from the maternal gut virome to her offspring. (Fig. 3). In contrast, cross-alignment of each mother dataset to phage contigs assembled from unrelated infants did not produce any common reads, thus indicating the absence of environmental contamination and supporting the notion of vertical transmission.

**Table 2:** List of viral taxa with abundance > 0.01% identified in the fecal samples of both mother and corresponding newborn

Viral taxonomy	Mother-infant 1	Mother-infant 2	Mother-infant 3	Mother-infant 4	Mother-infant 5	Mother-infant 6	Mother-infant 7
Unclassified__Bacillus virus 1						Shared	
Unclassified__Clostridium phage phiCT453A						Shared	
Unclassified__Geobacillus phage GBSV1						Shared	
Unclassified__Geobacillus virus E2					Shared	Shared	
Myoviridae Abouovirus__Brevibacillus virus Abouo						Shared	
Myoviridae Felixo1virus__Escherichia virus AYO145A	Shared	Shared	Shared	Shared		Shared	Shared
Myoviridae Felixo1virus__Escherichia virus EC6		Shared					Shared
Myoviridae Felixo1virus__Escherichia virus HY02							Shared
Myoviridae Felixo1virus__Escherichia virus JH2							Shared
Myoviridae Felixo1virus__Escherichia virus VpaE1							Shared
Myoviridae Felixo1virus__Salmonella virus FelixO1							Shared
Myoviridae Felixo1virus__Salmonella virus HB2014							Shared
Myoviridae Felixo1virus__Salmonella virus UAB87							Shared
Myoviridae Mooglevirus__Citrobacter phage Michonne		Shared					
Myoviridae Myoviridae_Unclassified__Bacillus phage 0305phi8-36						Shared	
Myoviridae Myoviridae_Unclassified__Bacillus phage AR9						Shared	
Myoviridae Myoviridae_Unclassified__Bacillus phage BCD7					Shared	Shared	
Myoviridae Myoviridae_Unclassified__Bacillus phage BM5						Shared	
Myoviridae Myoviridae_Unclassified__Bacillus phage G		Shared	Shared	Shared	Shared	Shared	Shared
Myoviridae Myoviridae_Unclassified__Bacillus phage SP-15			Shared		Shared	Shared	
Myoviridae Myoviridae_Unclassified__Brochothrix phage A9					Shared		
Myoviridae Myoviridae_Unclassified__Clostridium phage c-st					Shared	Shared	
Myoviridae Myoviridae_Unclassified__Clostridium phage phiCD211				Shared	Shared	Shared	
Myoviridae Myoviridae_Unclassified__Cronobacter phage vB_CsaM_GAP32			Shared	Shared	Shared	Shared	Shared
Myoviridae Myoviridae_Unclassified__Enterobacteria phage phi92			Shared	Shared		Shared	
Myoviridae Myoviridae_Unclassified__Escherichia phage vB_EcoM_Alf5							Shared
Myoviridae Myoviridae_Unclassified__Staphylococcus phage SA1							Shared
Unclassified__Paenibacillus phage phiIBB_Pl23						Shared	
Podoviridae Cba41virus__Cellulophaga virus Cba172					Shared		
Podoviridae Cp1virus__Streptococcus virus Cp1						Shared	
Podoviridae Phi29virus__Bacillus virus B103						Shared	
Podoviridae Phi29virus__Bacillus virus GA1					Shared	Shared	
Podoviridae Phi29virus__Bacillus virus phi29					Shared	Shared	
Podoviridae Podoviridae_Unclassified__Actinomyces phage Av-1					Shared	Shared	
Podoviridae Podoviridae_Unclassified__Bacillus phage Aurora						Shared	
Podoviridae Podoviridae_Unclassified__Bacillus phage MG-B1						Shared	
Podoviridae Podoviridae_Unclassified__Bacillus phage VMY22						Shared	
Podoviridae Podoviridae_Unclassified__Cellulophaga phage phi18:3					Shared		
Podoviridae Podoviridae_Unclassified__Planktothrix phage PaV-LD			Shared	Shared		Shared	
Podoviridae Podoviridae_Unclassified__Streptococcus phage Str-PAP-1	Shared						
Unclassified__Pseudomonas phage O4					Shared		
Siphoviridae C5virus__Lactobacillus virus c5					Shared		
Siphoviridae Cba181virus__Cellulophaga virus Cba181					Shared		
Siphoviridae Cecivirus__Bacillus virus 250					Shared		
Siphoviridae Ff47virus__Mycobacterium virus Ff47						Shared	
Siphoviridae Mudcatvirus__Arthrobacter virus Mudcat					Shared	Shared	
Siphoviridae Omegavirus__Mycobacterium phage Courthouse					Shared	Shared	
Siphoviridae Pepy6virus__Rhodococcus virus Pepy6	Shared				Shared	Shared	
Siphoviridae Pepy6virus__Rhodococcus virus Poco6						Shared	
Siphoviridae Phietavirus__Staphylococcus phage EW					Shared	Shared	
Siphoviridae Sfi21dt1virus__Streptococcus phage 7201				Shared			
Siphoviridae Sfi21dt1virus__Streptococcus phage Abc2				Shared			
Siphoviridae Sfi21dt1virus__Streptococcus phage DT1				Shared			
Siphoviridae_Unclassified__Bacillus phage BCJA1c	Shared						
Siphoviridae_Unclassified__Bacillus phage BtCS33						Shared	
Siphoviridae_Unclassified__Bacillus phage phi4J1				Shared	Shared	Shared	
Siphoviridae_Unclassified__Bacteriophage Lily	Shared						
Siphoviridae_Unclassified__Bacteroides phage B124-14					Shared		
Siphoviridae_Unclassified__Brevibacillus phage Sundance					Shared	Shared	
Siphoviridae_Unclassified__Cellulophaga phage phi46:1					Shared		
Siphoviridae_Unclassified__Clostridium phage 39-O						Shared	
Siphoviridae_Unclassified__Clostridium phage phi8074-B1					Shared		
Siphoviridae_Unclassified__Clostridium phage phiCT453B						Shared	
Siphoviridae_Unclassified__Croceibacter phage P2559Y					Shared		
Siphoviridae_Unclassified__Enterococcus phage EFC-1	Shared						
Siphoviridae_Unclassified__Geobacillus virus E3				Shared	Shared	Shared	
Siphoviridae_Unclassified__Helicobacter phage phiHP33					Shared		
Siphoviridae_Unclassified__Lactobacillus phage Ldl1					Shared		
Siphoviridae_Unclassified__Lactococcus phage 1706						Shared	
Siphoviridae_Unclassified__Lactococcus phage 50,101	Shared						
Siphoviridae_Unclassified__Lactococcus phage bIL285						Shared	Shared
Siphoviridae_Unclassified__Lactococcus phage Tuc2009							Shared
Siphoviridae_Unclassified__Mycobacterium phage BTCU-1						Shared	
Siphoviridae_Unclassified__Pseudomonas phage YMC11/07/P54_PAE_BP						Shared	
Siphoviridae_Unclassified__Riemerella phage RAP44					Shared		
Siphoviridae_Unclassified__Staphylococcus phage StB20					Shared		
Siphoviridae_Unclassified__Streptococcus phage Dp-1				Shared		Shared	
Siphoviridae_Unclassified__Streptococcus phage MM1	Shared					Shared	
Siphoviridae_Unclassified__Streptococcus phage PH15						Shared	
Siphoviridae_Unclassified__Streptococcus phage phiNJ2						Shared	
Siphoviridae_Unclassified__Streptococcus phage SM1	Shared					Shared	
Siphoviridae_Unclassified__Synechococcus phage S-CBS3					Shared		
Siphoviridae_Unclassified__Vibrio phage SIO-2						Shared	
Siphoviridae Spbetavirus__Bacillus virus SPbeta					Shared	Shared	
Unclassified__Streptococcus phage 20617			Shared	Shared	Shared	Shared	Shared
Unclassified__Streptococcus phage phiARI0131-2						Shared	
Unclassified__Uncultured phage crAssphage					Shared	Shared

### Genome decoding and functional characterization of vertically transmitted phage genomes

**Fig. 4:**
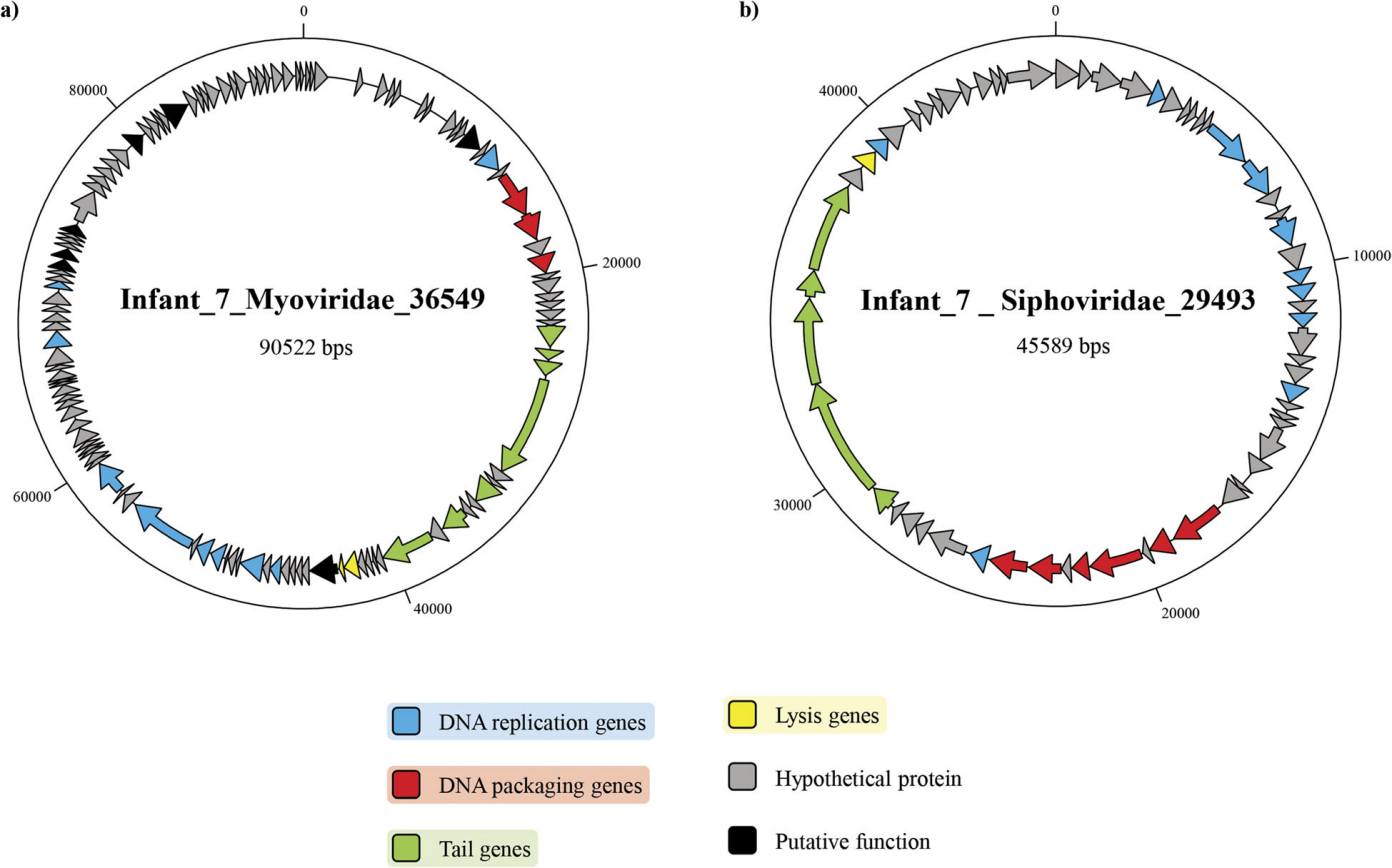
Genomic characterization of two vertically transmitted phages. **a**, **b** The genome map of the phages Infant_7_Myoviridae_36549 and Infant_7_Siphoviridae_29493, respectively. Genes are colored based on their predicted function

METAnnotatorX was employed for the reconstruction and functional characterization of complete viral genomes predicted to be transmitted from mother to newborn. This analysis resulted in the deduction of two phage genomes shared by Infant_7 and its corresponding mother’s phageome, named Infant_7_Myoviridae_36549 and Infant_7_Siphoviridae_29493, with genome sizes of 90,522 and 45,589 bp, respectively (Fig. 4). ORF prediction and functional annotation based on PHAST database [43] revealed that Infant_7_Myoviridae_36549 encodes 118 genes, 89 of which were shown to encode hypothetical proteins, while Infant_7_Siphoviridae_29493 encodes a total of 62 genes, representing 41 hypothetical proteins (Fig. 4). Interestingly, evaluation of the taxonomy of homologous genes identified in the PHAST database showed that 63% of the ORFs encoded by Infant_7_Myoviridae_36549 and 32% of the ORFs encoded by Infant_7_Siphoviridae_29493 share distant homology with genes encoded by *Bacillus* phage BCD7 and *Bacteroides* phage B124-14, respectively. This finding suggests that the hosts of Infant_7_Myoviridae_36549 and Infant_7_Siphoviridae_29493 are members of the Firmicutes and Bacteroidetes phyla.

Analysis of phage modules revealed that Infant_7_Myoviridae_36549 and Infant_7_ Siphoviridae_29493 possess four modules typical of *Myoviridae* and *Siphoviridae* phages, i.e., DNA replication, DNA packaging, and tail and lysis module (Fig. 4). Both phages lack a clear lysogeny module, with no genes encoding integrases found within the genomes (Fig. 4). Notably, Infant_7_Myoviridae_36549 possesses a large region encoding genes of unknown function interspersed with genes with putative functions such as a putative type III restriction protein and two queuosine (Que) biosynthesis genes. The latter compound is a modified nucleoside that is present in certain tRNAs [44, 45], and genes for its synthesis have been identified in other *Myoviridae* phages [46].

### Evaluation of the minimum amount of shotgun metagenomics data needed for robust phage biodiversity assessment

**Fig. 5:**
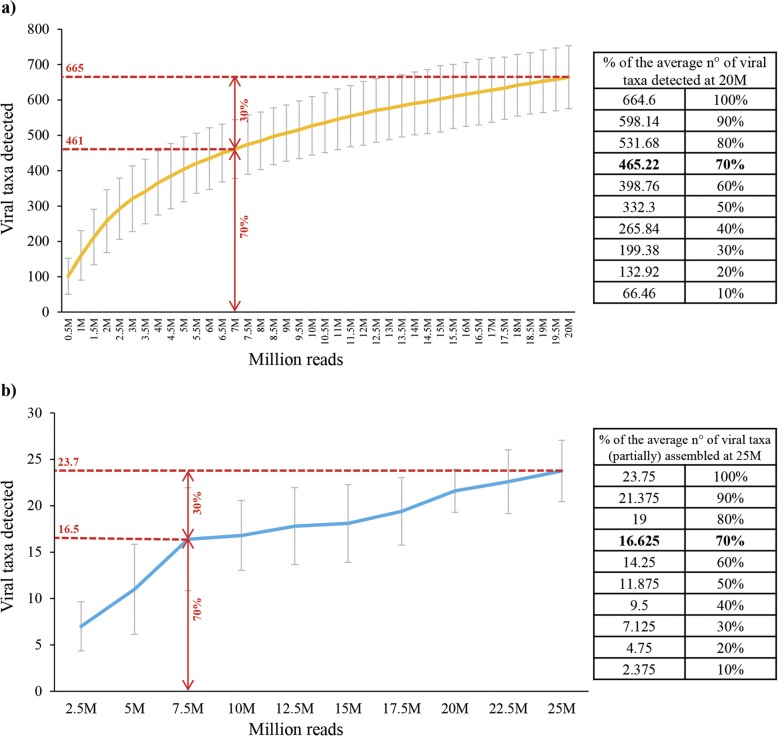
Evaluation of the optimal sequencing depth for read- and assembly-based analyses through investigation of five sequenced datasets. **a** The average number of viral taxa detected by means of read-based taxonomic profiling at increasing sub-samplings of the total read pools. **b** The average number of viral taxa detected among contigs assembled using increasing sub-samplings of the total read pools

The choice of the target sequencing depth is a critical step in resource management when planning phageome studies using shotgun metagenomics sequencing. To define the number of sequence reads needed to obtain a reliable and comprehensive coverage of the biodiversity from read- and contig-based analyses, the five datasets of mothers with > 20 M reads (Additional file 1: Table S4) were subjected to iterative analysis of subsamples to construct rarefaction curves reporting the number of phage species identified in sub-samplings from 0.5 M up to 20 M reads. Notably, for each of the five samples analyzed, the number of phage taxa detected increased exponentially until a read pool size of about 7 M reads, beyond which a plateauing was observed (Additional file 1: Figure S6). Moreover, the average curve obtained by integration of the five datasets revealed that 7.5 M reads are enough to cover 70% of the total biodiversity identified in the total pool of 20 M reads. This indicates that 7 M reads are the target sequencing depth needed to obtain a comprehensive read-based overview of the phage population harbored by a given fecal sample obtained from a healthy adult (Fig. 5).

Focusing on the assembly and analysis of phage genomes, we constructed a rarefaction curve reporting the number of viral taxa for which we obtained at least one assembled contig > 5000 bp at increasing subsampling points from 0.5 M up to 25 M reads. Interestingly, the obtained graphs revealed that the curve rapidly increased up to the point of 7.5 M reads and then tends to plateau (Additional file 1: Figure S6). Furthermore, evaluation of the average curve revealed that 7.5 M reads are enough to obtain contigs for 70% of the total number of phage taxa assembled 25 M reads.

Notably, evaluation of the logarithmic trendline for both the read- and contig-based rarefaction curves revealed that doubling the amount of shotgun metagenomic reads would only provide a limited increase of 14.4 and 16% in viral taxa identified through read profiling and contig classification, respectively (Additional file 1: Figure S7).

Altogether, these results indicate that the minimum sequencing depth needed for robust read-based profiling and assembly of gut phageomes of healthy adults is approximately 7.5 M reads. In fact, additional sequencing outputs do not provide additional valuable information about the biodiversity of phages in these complex ecosystems (Fig. 5). Nevertheless, re-evaluation and adjustment of the target sequencing depth is necessary in case of analysis of samples with remarkably lower or higher bacterial and viral biodiversity, e.g., infant gut or soil samples. In this context, we exploited the dataset of Infant 7 to reconstruct rarefaction curves of viral taxa observed trough taxonomic classification of reads and assembled contigs > 5000 bp (Additional file 1: Figure S8). Notably, these data confirmed 7 M reads as an optimal sequencing depth also for comprehensive analysis of infant phageomes (Additional file 1: Figure S8).

## Conclusions

Despite environmental and host-associated microbiomes being the subject of an increasing number of studies, the phageome associated with these complex bacterial communities remains poorly understood. This is primarily due to the current lack of a gold standard procedure for viral DNA extraction and data analysis. Instead, there are a variety of different procedures associated with publications, which makes it near impossible to compare results between different studies.

To address this issue, we performed a comparative assessment of various DNA extraction methods for virome analysis and developed a novel bioinformatic tool, METAnnotatorX, which enables an integrated and comprehensive processing of viral and prokaryotic metagenomic datasets. Notably, this software can perform a wide range of read- and assembly-based analyses and represents, to date, the most complete bioinformatics platform for the study of viromes. METAnnotatorX was employed to perform an in-depth comparison of five protocols for viral DNA extraction and enrichment, leading to the identification of protocol 1E as the one that performs best in terms of removal of non-viral DNA, unbiased representation of the viral population and execution time. Moreover, we also analyzed five deep-sequenced viromes retrieved from feces of human adults. The generated results demonstrated that 7.5 M reads represent a sufficient sequencing depth needed for both read- and assembly-based investigation of gut phageomes of heathy human adults.

The proposed comprehensive pipeline for phageome analysis was then used to shed light on the vertical acquisition of phages by infants. Analysis of fecal samples collected from seven mothers and their newborns revealed that they share identical phage genotypes, thus indicating the existence of a putative vertical route for transmission of phages from the mother to the infant. Moreover, METAnnotatorX also allowed, for the first time, the reconstruction and characterization of the genome of two genotypes predicted to be vertically transmitted.

Notably, these results demonstrate that the use of a comprehensive pipeline for analysis of phageomes will be pivotal for future explorations of the dark matter of phageomes, such as phage ecology in the gut environment, the role of phages in modulating the bacterial population and their impact on the physiology as well as bacterial community kinetics as players of dysbiosis and homeostasis in the gut microbiota.

## Additional files

Additional file 1: Supplementary text, tables and figures. (DOCX 5306 kb)

Additional file 2: Archaeal and bacterial viruses profiled in the analyzed samples. (XLSX 152 kb)
