# Supplementary material for: Tracing mother-infant transmission of bacteriophages by means of a novel analytical tool for shotgun metagenomic datasets: METAnnotatorX
Source: Microbiome. 2018 Aug 20;6:145. doi: 10.1186/s40168-018-0527-z (PMC6102903; doi:10.1186/s40168-018-0527-z)

**SUPPLEMENTARY TEXT**

**Comparison with available tools for phageome analysis.**

At the time of writing, VIROME (1) and MetaVir 2 (2) are the only available tools that are specifically developed for analysis of phageome datasets. However, VIROME works only on a maximum of 250,000 long reads (> 300 bp) and does not allow the analysis of Illumina datasets, while the web server of MetaVir 2 is unavailable due to a saturation of storage space and computing capacities. Due to these limitations, we were not able to compare METAnnotatorX with other read- or assembly-based tools for analysis of prokaryotic metaviromes.

Nevertheless, analysis of VIROME and MetaVir 2 revealed that, in contrast to METAnnotatorX, they do not allow the assignment of reads to Archaea, Bacteria and Eukaryote. Moreover, METAnnotatorX is the only tool that offers a range of integrated additional analyses, which include functional classification of reads as well as contig assembly, annotation, cataloguing by taxonomy, functional profiling and discovery of novel phage taxa.

**References**

1. Wommack, K.E., Bhavsar, J., Polson, S.W., Chen, J., Dumas, M., Srinivasiah, S., Furman, M., Jamindar, S. and Nasko, D.J. (2012) VIROME: a standard operating procedure for analysis of viral metagenome sequences. *Stand Genomic Sci*, **6**, 427-439.

2. Roux, S., Tournayre, J., Mahul, A., Debroas, D. and Enault, F. (2014) Metavir 2: new tools for viral metagenome comparison and assembled virome analysis. *BMC Bioinformatics*, **15**, 76.

**Tables**

| **Table S1**. METAnnotatorX’s setting variables. |  |
| --- | --- |
| **Parameter** | **Value** |
| Number of threads used by the pipeline | **64** |
|  |  |
| Read based analyses | |
| Minimum remaining sequence length (fastq-mcf) | **100** |
| Quality threshold causing base removal (fastq-mcf) | **20** |
| sKew percentage-less-than causing cycle removal (fastq-mcf) | 0 |
| Minimum mean quality score (fastq-mcf) | 20 |
| Window-size for quality trimming (fastq-mcf) | 5 |
| Maximum number of reads | **5,000,000** |
| Viral e-value cutoff (rapsearch) | **4** |
| Viral alignment minimum length (rapsearch) | **20** |
| Archaea e-value cutoff (rapsearch) | **15** |
| Archaea alignment minimum length (rapsearch) | **20** |
| Bacteria e-value cutoff (rapsearch) | **4** |
| Bacteria alignment minimum length (rapsearch) | **20** |
| Eukaryota e-value cutoff (rapsearch) | **4** |
| Eukaryota alignment minimum length (rapsearch) | **20** |
| Cluster of Orthologous Genes e-value cutoff (rapsearch) | 2 |
| Glycobiome e-value cutoff (rapsearch) | 2 |
| Pathways e-value cutoff (rapsearch) | 2 |
|  |  |
| Contig based analyses | |
| Reads technology (SPAdes) | **paired** |
| Reads length (SPAdes) | **250** |
| K-mer sizes to be used (SPAdes)* | 21,33,55,77,99,127 |
| Assembly method (SPAdes) | --meta |
| Minimum contig length | **5000** |
| Minimum Taxonomy cutoff | **10 %** |
| Viral e-value cutoff (rapsearch) | **4** |
| Viral alignment minimum length (rapsearch) | **20** |
| Archaea e-value cutoff (rapsearch) | **20** |
| Archaea alignment minimum length (rapsearch) | **20** |
| Bacteria e-value cutoff (rapsearch) | **4** |
| Bacteria alignment minimum length (rapsearch) | **20** |
| Eukaryota e-value cutoff (rapsearch) | **4** |
| Eukaryota alignment minimum length (rapsearch) | **20** |
| Gene annotation e-value cutoff (rapsearch) | **5** |
| Gene annotation alignment minimum length (rapsearch) | **20** |
| Cluster of Orthologous Genes e-value cutoff (rapsearch) | **5** |
| COG genus minimum length (rapsearch) | **20** |
| Glycobiome e-value cutoff (rapsearch) | **5** |
| Glycobiome genus minimum length (rapsearch) | **20** |
| Pathways e-value cutoff (rapsearch) | **5** |
| Pathways genus minimum length (rapsearch) | **20** |
| CRISPR e-value cutoff (rapsearch) | 2 |
| *calculated by employing read length. | |
| **Values indicated in bold** can be modified by the user in the parameters file. | |

| **Table S2: Re-analysis of datasets from Minot et al., 2012, PLoS One processed in the MetaVir2 website.** | | | |
| --- | --- | --- | --- |
| **Sample** |  | **MetaVir2** | **METAnnotatorX** |
| **Human gut - Subject 1** | **n° of processed sequences** | 3638 | 3638 |
|  | **n° of detected viral taxa** | 556 | 675 |
| **Human gut - All subjects** | **n° of processed sequences** | 10202 | 10202 |
|  | **n° of detected viral taxa** | 933 | 1186 |

| **Table S3:** Composition and analysis of the artificial sample. | | |
| --- | --- | --- |
|  | **Artificial dataset composition** | **Observed composition** |
| Gut Virome (Human Adult) | 63.89% | 65.60% |
| *Lactococcus lactis* phage c2 | 35.00% | 33.45% |
| *Lactococcus lactis* phage 936 | 1.11% | 0.94% |

| **Table S4:** Table of quality-filtered shotgun metagenomics reads sequenced. | | | | | | | |
| --- | --- | --- | --- | --- | --- | --- | --- |
| **Sample** | **Infant 1** | **Infant 2** | **Infant 3** | **Infant 4** | **Infant 5** | **Infant 6** | **Infant 7** |
| **n° of Quality-Filtered reads** | 7543885 | 238288 | 645416 | 864252 | 17020366 | 11303968 | 34105775 |
|  |  |  |  |  |  |  |  |
| **Sample** | **Mother 1** | **Mother 2** | **Mother 3** | **Mother 4** | **Mother 5** | **Mother 6** | **Mother 7** |
| **n° of Quality-Filtered reads** | 25434271 | 5118701 | 4002202 | 4904160 | 28175832 | 25860303 | 8666322 |

**Supplementary Figures legends**

**Figure S1:** The METAnnotatorX Graphical User Interface (GUI). Panel a displays, starting from the top, the screenshot of the necessary files in the working directory, the command to be executed in the Unix shell, the interface for selection of the project name and the interface for selection of input files. Panel b shows an extract of the file “parameters.txt” that includes all parameters used by METAnnotatorX to perform the metagenomic analyses.

**Figure S2:** Evaluation of non-viral DNA removal performances through analysis of viral DNA extracted in duplicates from the same faecal sample using five different protocols. The bar plot reports the percentage of viral, bacterial, archaeal and eukaryotic DNA detected through taxonomic classification of reads corresponding to coding regions.

**Figure S3:** MAUVE genomic alignment of contigs assembled from phageome datasets obtained from meconium samples. Panel a and b show alignment of contigs classified as Siphoviridae and Myoviridae phages, respectively.

**Figure S4:** MAUVE genomic alignment of contigs assembled from 12 publicly available infant phageomes. The image reports two portions of the genomic alignment obtained with contigs assembled from the 12 publicly available infant phageomes. Contigs shared by most of the datasets are highlighted in red.

**Figure S5:** Taxonomic profiling of seven mothers sampled at 34 weeks of gestation and meconium samples of their corresponding offspring. The predicted viral, archaeal, bacterial and eukaryotic relative read abundance is reported through a bar plot for each mother and infant.

**Figure S6:** Viral taxa observed from read- and assembly-based analyses through investigation of five datasets. Panel a reports the number of viral taxa detected by means of read-based taxonomic profiling at increasing sub-samplings of the five reads pools analyzed. Panel b represents the number of viral taxa detected among contigs assembled using increasing sub-samplings of the five reads pools analyzed.

**Figure S7:** Trendline predicted for read- and contig-based curves of observed viral taxa. Panel a and b reports the logarithmic trendline predicted for the curve reporting the number of viral taxa detected by means of read-based and contig-based taxonomic profiling at increasing sub-samplings of the five reads pools analyzed.

**Figure S8.** Evaluation of the optimal sequencing depth for read- and assembly-based analyses of infant-derived fecal phageome datasets. Panel a reports the average number of viral taxa detected by means of read-based taxonomic profiling at increasing sub-samplings of the total read pool. Panel b represents the average number of viral taxa detected among contigs assembled using increasing sub-samplings of the total read pool.

**
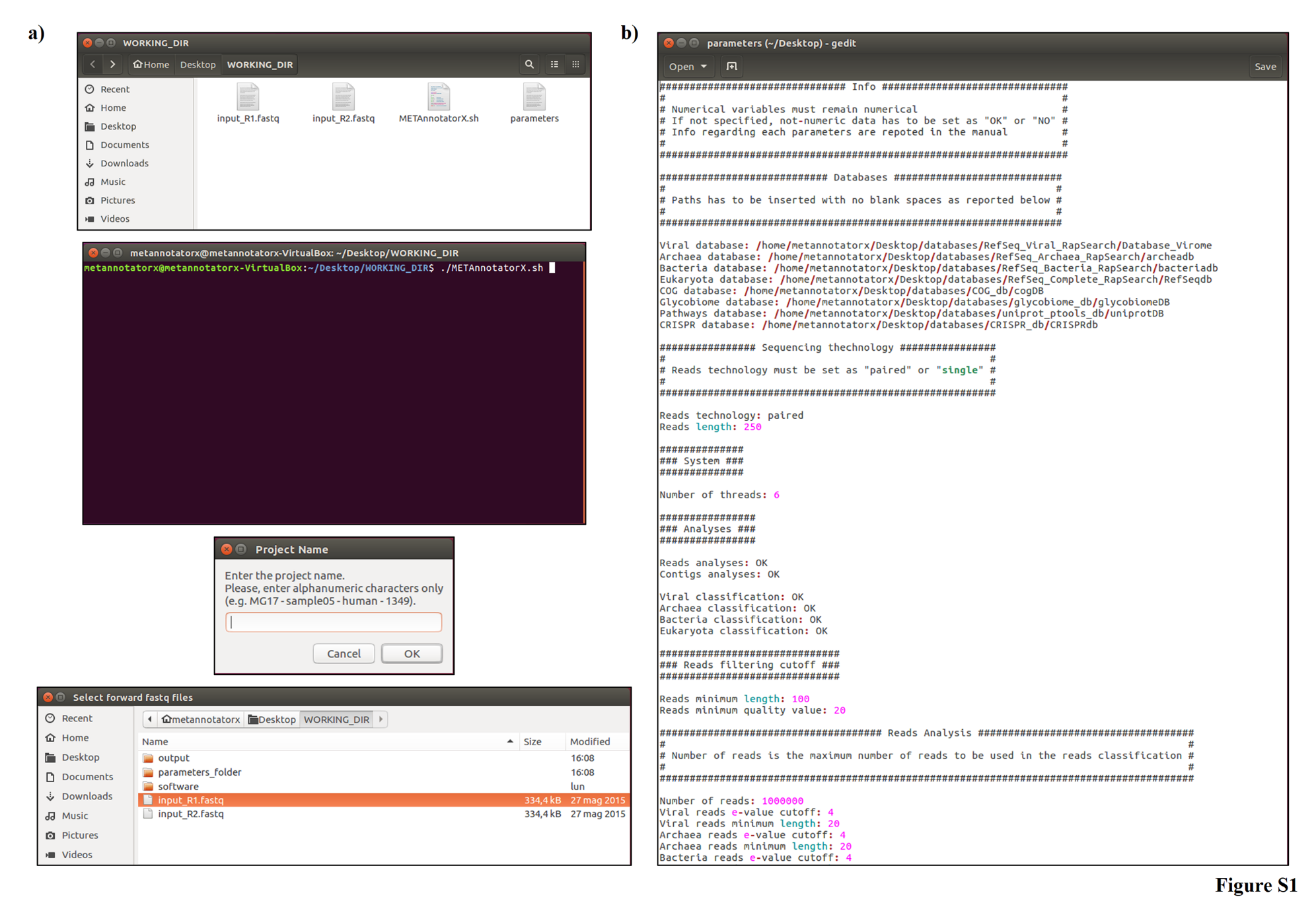
**


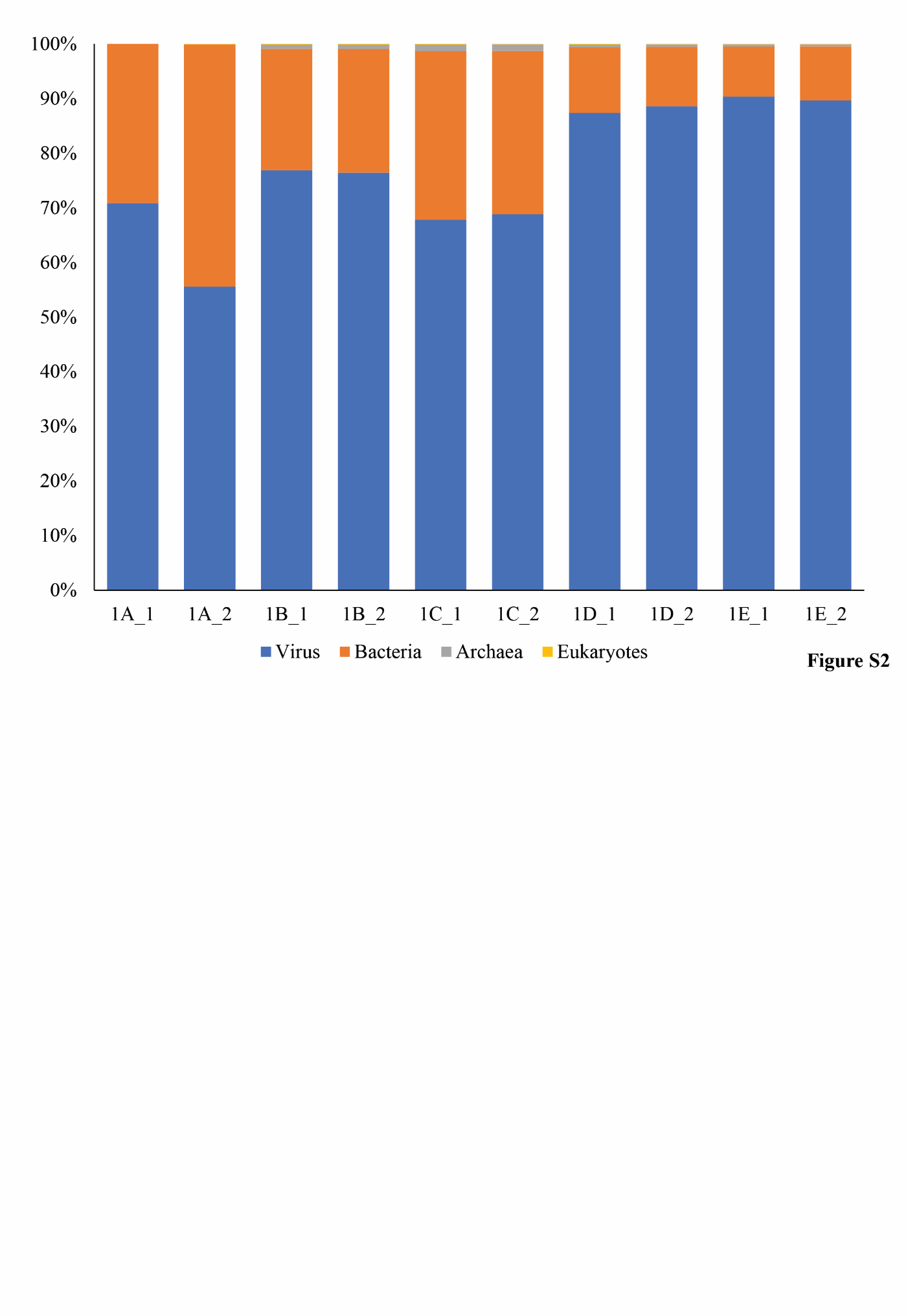


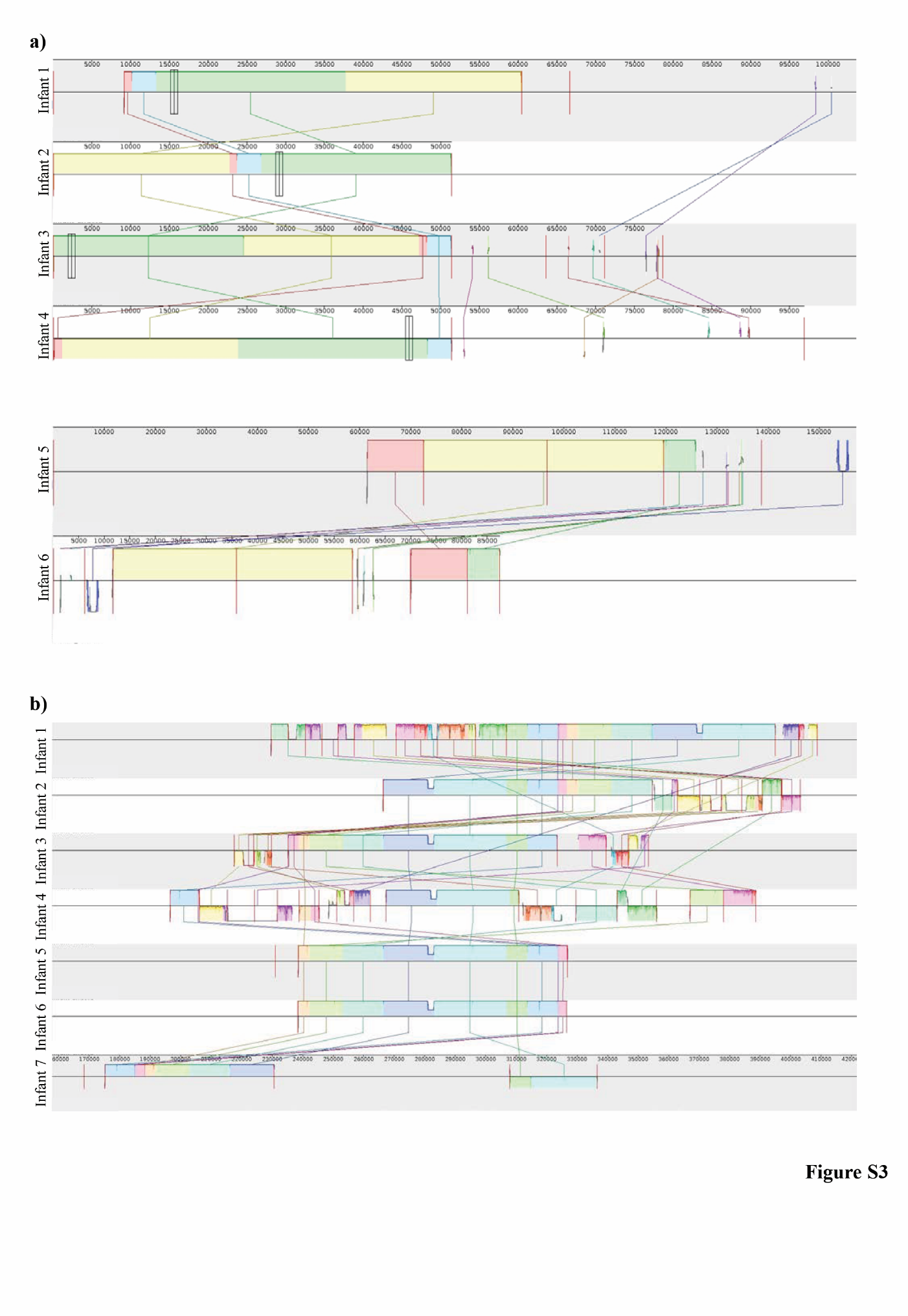


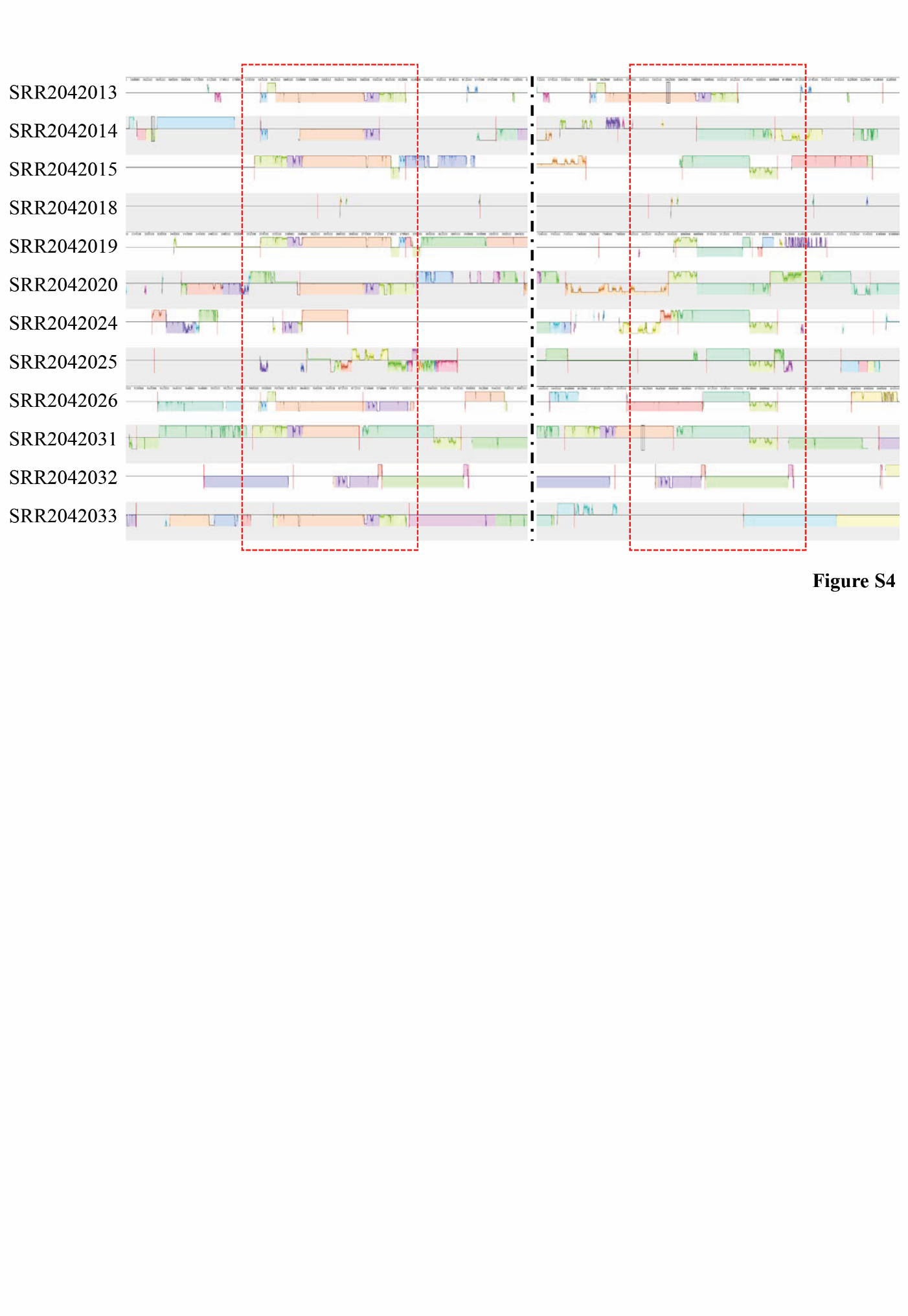


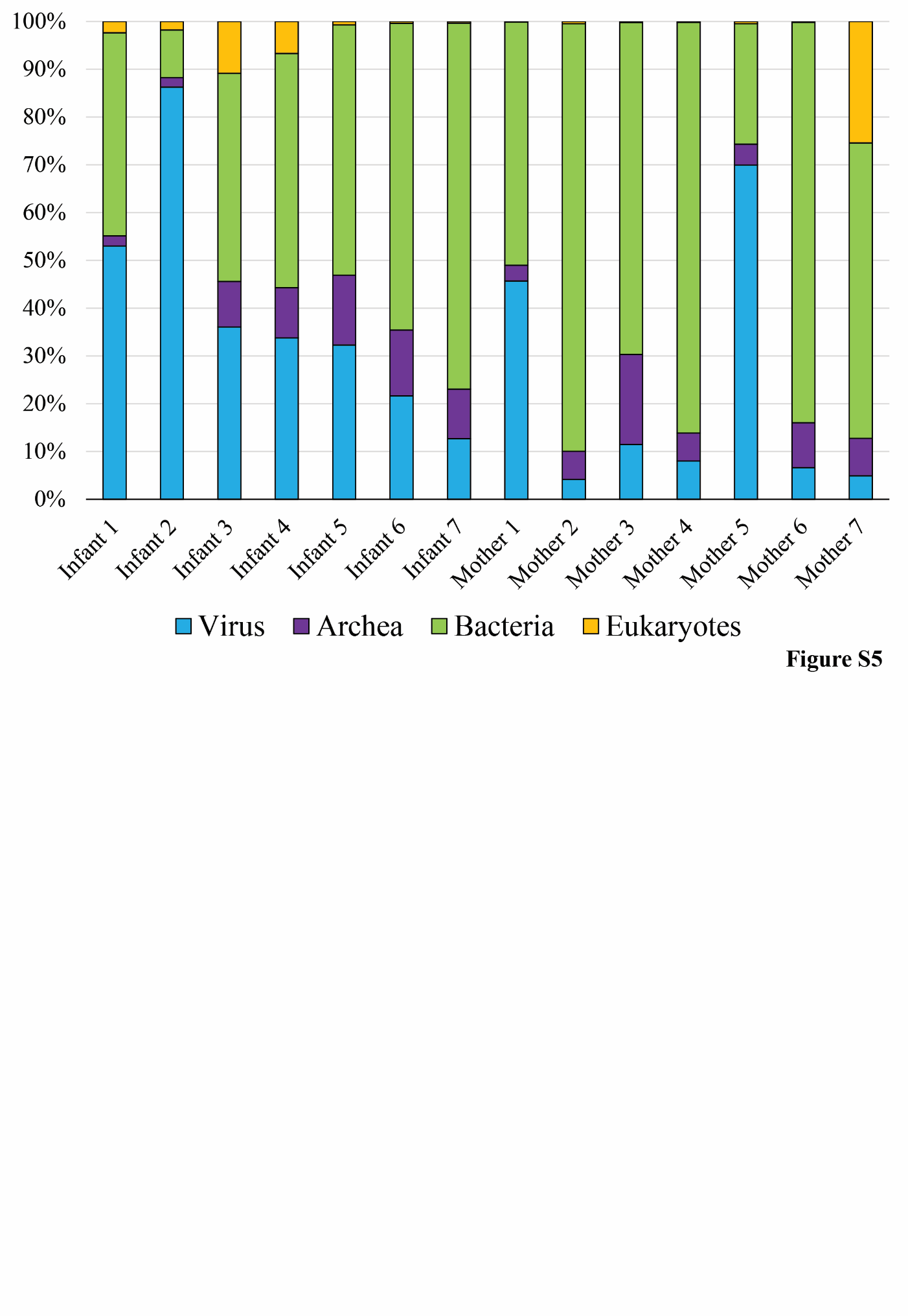


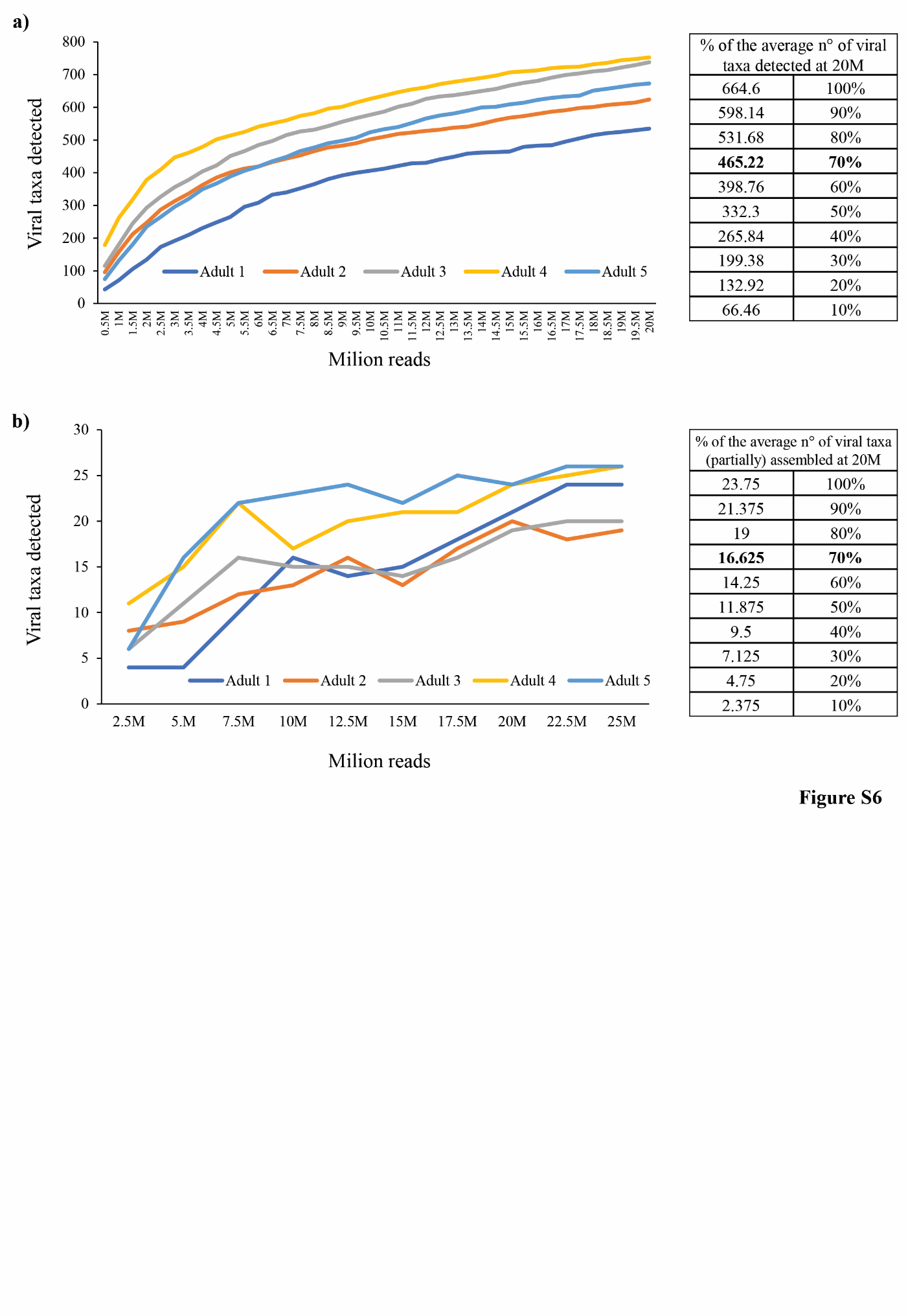


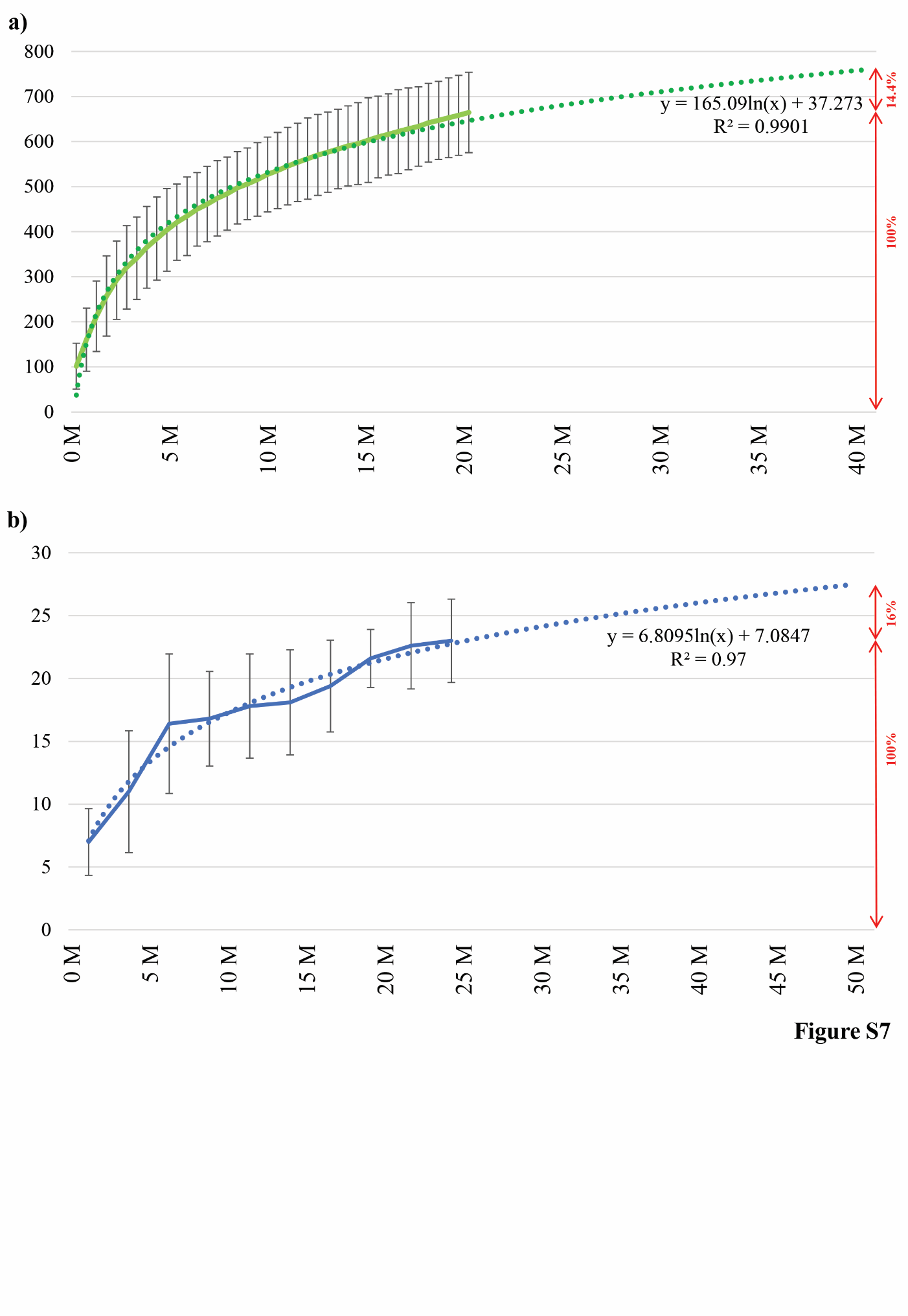


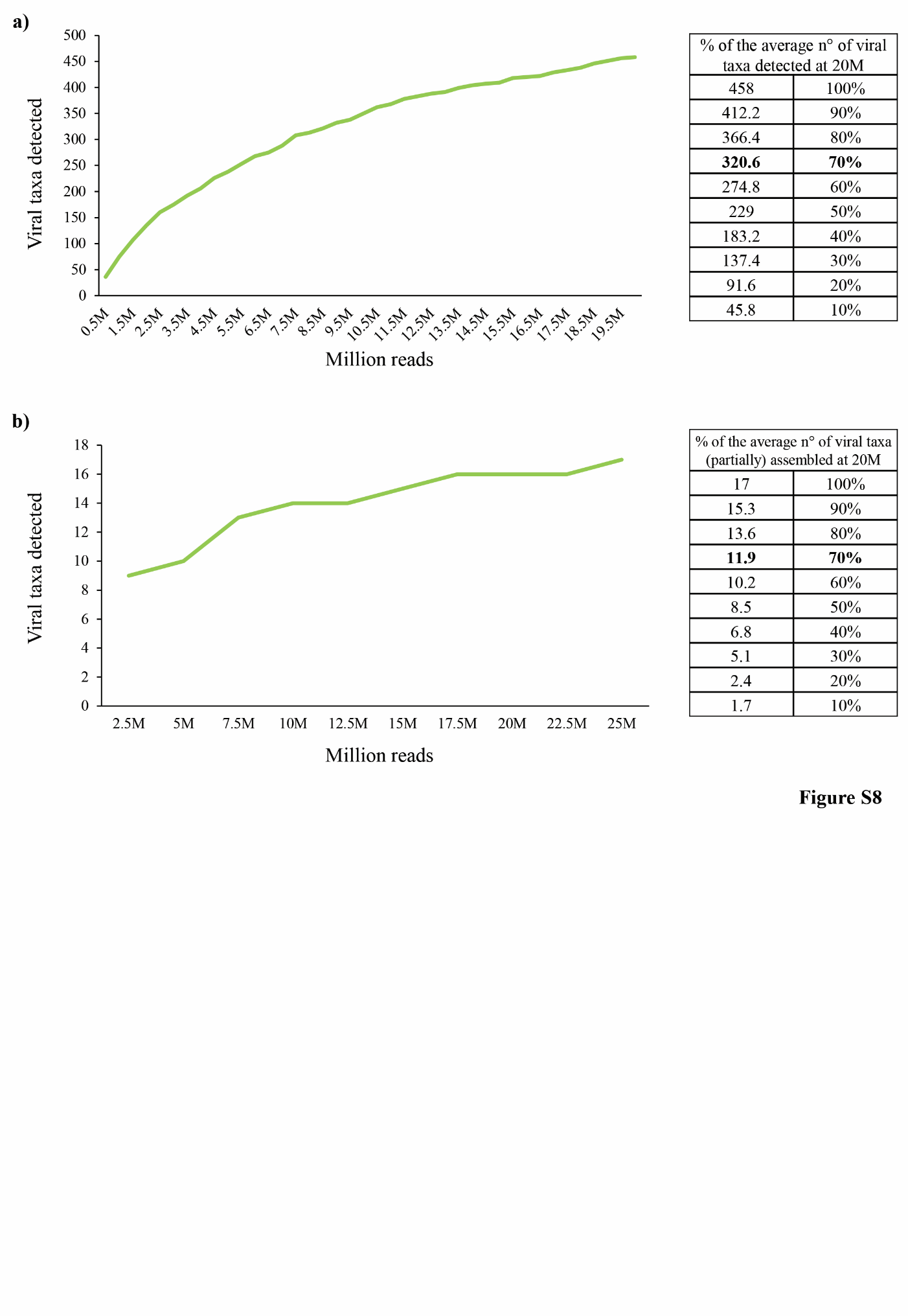

Supplement: Supplementary file 1 — Supplementary text, tables and figures. (DOCX 5306 kb) [file 40168_2018_527_MOESM1_ESM.docx]
